# Expression of CD64 on Circulating Neutrophils Favoring Systemic Inflammatory Status in Erythema Nodosum Leprosum

**DOI:** 10.1371/journal.pntd.0004955

**Published:** 2016-08-24

**Authors:** Veronica Schmitz, Rhana Berto da Silva Prata, Mayara Garcia de Mattos Barbosa, Mayara Abud Mendes, Sheila Santos Brandão, Thaís Porto Amadeu, Luciana Silva Rodrigues, Helen Ferreira, Fabrício da Mota Ramalho Costa, Jessica Brandão dos Santos, Fabiana dos Santos Pacheco, Alice de Miranda Machado, José Augusto da Costa Nery, Mariana de Andrea Hacker, Anna Maria Sales, Roberta Olmo Pinheiro, Euzenir Nunes Sarno

**Affiliations:** 1 Instituto Oswaldo Cruz, Fundação Oswaldo Cruz, Rio de Janeiro, Brazil; 2 Faculdade de Ciências Médicas, Universidade do Estado do Rio de Janeiro, Rio de Janeiro, Brazil; Fondation Raoul Follereau, FRANCE

## Abstract

Erythema Nodosum Leprosum (ENL) is an immune reaction in leprosy that aggravates the patient´s clinical condition. ENL presents systemic symptoms of an acute infectious syndrome with high leukocytosis and intense malaise clinically similar to sepsis. The treatment of ENL patients requires immunosuppression and thus needs to be early and efficient to prevent both disabilities and permanent nerve damage. Some patients experience multiple episodes of ENL and prolonged use of immunosuppressive drugs may lead to serious adverse effects. Thalidomide treatment is extremely effective at ameliorating ENL symptoms. Several mechanisms have been proposed to explain the efficacy of thalidomide in ENL, including the inhibition of TNF production. Given its teratogenicity, thalidomide is prohibitive for women of childbearing age. A rational search for molecular targets during ENL episodes is essential to better understand the disease mechanisms involved, which may also lead to the discovery of new drugs and diagnostic tests. Previous studies have demonstrated that IFN-γ and GM-CSF, involved in the induction of CD64 expression, increase during ENL. The aim of the present study was to investigate CD64 expression during ENL and whether thalidomide treatment modulated its expression. Leprosy patients were allocated to one of five groups: (1) Lepromatous leprosy, (2) Borderline leprosy, (3) Reversal reaction, (4) ENL, and (5) ENL 7 days after thalidomide treatment. The present study demonstrated that CD64 mRNA and protein were expressed in ENL lesions and that thalidomide treatment reduced CD64 expression and neutrophil infiltrates—a hallmark of ENL. We also showed that ENL blood neutrophils exclusively expressed CD64 on the cell surface and that thalidomide diminished overall expression. Patient classification based on clinical symptoms found that severe ENL presented high levels of neutrophil CD64. Collectively, these data revealed that ENL neutrophils express CD64, presumably contributing to the immunopathogenesis of the disease.

## Author Summary

Leprosy reactions are an acute exacerbation of a patient´s clinical condition. Reactions are classified into type 1 (reversal reaction; RR) and type 2 (erythema nodosum leprosum; ENL) according to the etiopathogenesis. Early detection of both types of reactional states is fundamental to treatment management with the most adequate available drugs to ameliorate symptoms and avoid permanent disabilities. The current study investigated whether or not CD64 is expressed during ENL. Analyses of circulating neutrophils revealed that ENL patients expressed higher levels of surface CD64 in comparison to those with nonreactional leprosy and that the severity of ENL was coupled with high levels of CD64 expression. Despite the limited number of patients included in this study, it demonstrated that measurement of neutrophil CD64 expression could be used as a prognostic biomarker of ENL and that quantifying the CD64 response could also help indicate the severity of ENL. Indeed, the methodology adopted found that circulating neutrophil CD64 expression could provide a rapid and non-invasive ENL diagnosis capable of detecting reactions in outpatient clinics as well as leprosy reference centers, leading to more effective therapeutic decisions.

## Introduction

Leprosy, the leading infectious cause of disability, is a chronic infectious disease caused by *Mycobacterium leprae*. The disease is characterized by skin lesions and peripheral nerve impairment reflecting the preference of the bacteria for macrophages and Schwann cells. The number of new case detections during 2013, as reported by 105 countries, was 219,075 while the registered global prevalence at the end of that same year was 181,618 [1].

Leprosy is manifested by a spectrum of clinical and histopathological presentations that are directly related to the immune status of the patient. One pole of the disease, known as polar tuberculoid leprosy (TT), is characterized by an efficient specific cellular immune response. In contrast, the absence of an immune response with no apparent resistance to *M*. *leprae* characterizes lepromatous leprosy (LL) at the opposite pole. Most affected individuals show intermediate clinical and immunological patterns, commonly referred to as borderline tuberculoid (BT), borderline borderline (BB), and borderline lepromatous (BL) [2,3].

While the number of new cases has declined in recent years, leprosy remains a major public health challenge in the affected countries, mainly due to the sudden appearance of reactional forms. Leprosy reactions are an acute exacerbation of a patient’s clinical condition. Reactions are classified as either type 1 (reversal reaction; RR) or type 2 (erythema nodosum leprosum; ENL) according to the existing etiopathogenesis. Early detection of these reactional states is fundamental to adequately managing the disease with the drugs at hand to ameliorate symptoms and avoid permanent disabilities.

ENL is observed in up to 50% of all lepromatous leprosy patients and may occur at any time during the course of the disease, even in those considered cured [4–7]. ENL affects the skin and other organs and frequently presents systemic symptoms of the acute infections syndrome with high leukocytosis levels and intense malaise clinically similar to sepsis [8,9]. For several years, it was assumed that the main mechanism involved in ENL was the deposition of the immune complex, as evidenced by granular deposits of immunoglobulin and complement in perivascular [10] and extravascular sites, detection of immune complexes in vessel walls, and injured endothelial cells [11]. Recent data, however, suggest that the clinical course of ENL is correlated to the production of cytokines and pro-inflammatory mediators in the lesion sites or their systemic release [12–14]. Therefore, the inflammatory reaction would result from a complex combination of humoral and cellular factors of inflammation. The changes associated with the classic histopathology of acute ENL include the presence of an inflammatory infiltrate of polymorphonuclear leukocytes (PMN) in the deep layers of the dermis and subcutaneous tissue, frequently accompanied by a cluster of macrophages [15,16].

In an effort to facilitate rapid diagnostic approaches, the **s**earch for biomarkers is essential to achieving a better understanding of the molecular and cellular mechanisms underlying ENL. In this context, PMN may function as biological sensors because surface antigens undergo a number of changes during neutrophilic maturation. Thus, a rapid blood test capable of detecting such variation would be useful for predicting an ENL reactional episode.

CD64 (FcγRI), expressed in large amounts by mononuclear phagocytes, is a high-affinity receptor for monomeric IgG1 and IgG3. Resting PMN express very low levels of CD64 [17]. Nonetheless, the expression of this biomarker is directly upregulated by interferon-gamma (IFN-γ) and granulocyte colony-stimulating factor (G-CSF) acting on precursor cells in bone marrow [18,19]. The biological effects of CD64 activation lead to the internalization of immune complexes, degranulation, activation of the oxidative burst, and cytokine release [20,21]. Moreover, CD64 binds C-reactive protein (CRP) [22] and serum amyloid-P [23].

An increase in PMN-CD64 surface expression is observed in certain bacterial infections [24] so that it has been proposed as an improved diagnostic test for the evaluation of systemic inflammation and tissue injury [25]. Besides, CD64-expressing neutrophils have been used to distinguish patients with infection from those undergoing inflammatory states such as rheumatoid arthritis and systemic lupus erythematosus [26,27]. Interestingly, PMN CD64 expression is an early marker of severity and outcome in sepsis [28], being associated with the severity and prognosis of disseminated intravascular coagulation [29].

Previous studies have revealed that IFN-γ and GM-CSF, formal inducers of CD64 [18, 19], are expressed in the skin lesions and blood of ENL patients [30,31]. Additionally, activation of CD64 leads to the production of the inflammatory mediators observed during the emergence of ENL. The characterization of neutrophil CD64 expression across the leprosy spectrum could help identify an ENL patient who could benefit from early intervention. As such, the aims of this study were to examine neutrophil CD64 expression in leprosy patients with ENL and to evaluate the clinical relevance of CD64 quantification.

## Methods

### Patient information

This case-control based study was conducted at the Souza Araújo Outpatient Clinic, a reference center for leprosy diagnosis and treatment (Leprosy Laboratory, Oswaldo Cruz Foundation, Rio de Janeiro, RJ, Brazil). Leprosy had been diagnosed in individuals who had hypopigmented, anaesthetic skin patches and/or thickened nerves and/or acid-fast bacilli on their slit skin smears. Leprosy patients were classified according to the Ridley and Jopling scale [2] using clinical, histological, and bacteriological indices. The present study was approved by the Ethics Committee of the Oswaldo Cruz Foundation (CAAE 24006713.7.0000.5248). Informed written consent was obtained from all individuals included in the study and from parents of all under-aged participants. Patient recruitment took place during 2009–2015. The study population was divided into two groups: Group I: Health volunteers (n = 16; 7 females and 9 males, ranging in age from 24 to 56, matched for sex, age and locality with the patients group. Group II: Leprosy patients (n = 62; 46 males, 16 females, in a 17–72 age range). Patient clinical data are presented in S1–S6 Tables. The case definition of ENL was defined as a patient diagnosed with leprosy that had an acute appearance of crops of tender cutaneous or subcutaneous lesions, accompanied or not by fever, malaise, or other systemic involvement. The case definition of RR was a patient diagnosed with leprosy presenting acute inflammation of pre-existing leprosy lesions and/or the onset of new erythematous skin lesions.

### ENL severity scale

The clinical classification of mild, moderate, and severe ENL was ascertained in accordance with a patient´s clinical symptomatology [32–35]. Briefly, those classified with mild ENL had less than 10 tender skin lesions, most often located in only a few regions but without systemic signs or symptoms. Moderate ENL included patients presenting from 10-to-20 nodules ranging from painful to palpation in association with a moderate fever (<38.4°C) and discreet systemic symptomatology possibly affecting local and/or regional lymph node chains. A patient with **s**evere ENL demonstrated more than 20 spontaneously painful nodules, with possibly vesicular or ulcerated lesions often involving a large area of tegument (more than 5 regions) accompanied by expressive systemic symptomatology like high fever (>38.5°C), arthralgia, chills, chronic headaches, anorexia, fatigue, and overall involvement of lymph node chains.

### Clinical specimens and sample collection

Whole blood heparin samples were collected from all leprosy patients and healthy donors enrolled in the study. Male thalidomide-treated ENL patients had a second time point 7 days after initiating therapy (300 mg/day). All patients responded well to treatment, the majority experiencing a complete resolution of their cutaneous lesions within 7 days. Heparinized whole blood was used for flow cytometry analysis. Whole blood was collected directly into 2.5 mL PAXgene blood RNA tubes (Qiagen, Mississauga, Canada) for quantitative RT-PCR, and stored at -70°C.

Skin biopsy specimens (6 mm in diameter) containing both epidermis and dermis were taken from an active skin lesion at enrollment for diagnosis. If the patient developed ENL, a second skin biopsy was taken 7 days after beginning thalidomide treatment. Skin samples were divided in half: one portion was fixed in 10% buffered formalin and the other, snap frozen in liquid nitrogen for histopathological, immunohistochemical, RT-qPCR, and immunoblotting analyses.

### Histopathology

The skin biopsies were processed and embedded in paraffin and serially sectioned in the sagittal plane at 5 μm thickness on a Leica microtome. Sections were stained with Haematoxylin and Eosin stain (H&E stain) to study morphology, and modified Fite Wade´s stain to identify acid fast bacilli (AFB). AFB was graded according to Ridley scale of 0 to 6+ as Bacillary Index (BI). Images were obtained via Nikon Eclipse microscope with Infinity Capture software.

### Immunohistochemical staining

ENL frozen skin sections (4 μm) were analysed by the immunoperoxidase technique, as previously described [36]. Briefly, mouse IgG1 anti-human CD64 antibody (1:50, Biolegend, catalog number 305002) was diluted in PBS and incubated for 1 h at room temperature. The sections were washed 3 times and incubated with biotinylated horse anti-mouse IgG (VECTASTAIN Elite ABC Kit Mouse IgG) for 1 h at room temperature. After washing, the sections were incubated for 40 min with avidin–biotin complex (VECTASTAIN Elite ABC Kit Mouse IgG) for signal amplification, washed, and then incubated with substrate 3-amino-9-ethylcarbazole for 10 min (AEC Peroxidase HRP Substrate Kit, Vector Laboratories; catalog number SK-4200). Slides were counterstained with Mayer’s haematoxylin and mounted with aqueous faramount mounting medium (Dako, catalog number S3025). Images were obtained using Nikon Eclipse E400 microscope Infinity Capture software.

### Immunofluorescence assay

Frozen ENL skin lesion sections assays were performed in a Leica LM3000 cryostat, fixed in acetone, and hydrated in 0.01 M PBS. Unspecific binding sites were blocked with 10% Normal Goat Serum (NGS; Sigma-Aldrich) and 5% Fetal Calf Serum (FCS, GIBCO, Life Technologies) in 0.01 M PBS for 1 h at room temperature. Mouse IgG1 anti-human CD64 (1:50; Biolegend, 305002) and rabbit IgG anti-human MPO (1:100; Santa Cruz Biotechnology, SC-16128-R) and their respective isotypes were diluted in 1% Bovine Serum Albumin (BSA, Sigma-Aldrich), 1% NGS in 0.01 M PBS and incubated at 4°C overnight. Tissue sections were washed 3 times and incubated with Alexa Fluor 532 goat anti-mouse IgG (1:1000, ThermoFisher Scientific, A-11002) and Alexa Fluor 633 goat anti-rabbit IgG secondary antibodies (1:1000, ThermoFisher Scientific, A-21070) for 1:30 h at room temperature. The nuclei were stained with 4′-6-diamidino-2-phenylindole (DAPI; 1:10000, Molecular Probes, D1306), and slides were mounted with VECTASHIELD Mounting Medium (Vector Laboratories, H-1000). Tissues were imaged using an Axio Observer.Z1 fluorescence microscope equipped with a Colibri.2 illumination system (Carl Zeiss, Oberkochen, Germany) and the EC Plan-Neofluar 20x/0.50 objective and Plan-Apochromat 63x/1.3 oil objective. Images were acquired with a digital camera AxioCam HRm and AxioVision Rel. 4.6 software (Carl Zeiss).

### Flow cytometry

Expression of CD64 was measured via the FACSCalibur flow cytometer (Becton Dickinson, NY, USA) using the Leuko64 test kit (Trillium Diagnostic, Brewer, ME, USA). According to the manufacturer’s instruction, 50μL heparin-anticoagulated whole blood was incubated for 10 minutes in the dark at room temperature with a mixture of murine monoclonal antibodies: anti-CD64 (fluorescein isothiocynate (FITC) conjugated clones 22 and 32.2) or anti-CD64 CD163 (phycoerythrin (PE) conjugated clone Mac2-148) followed by red cell lysis solution (Trillium Lyse). Fluorescence beads were then added and flow cytometer analysis was performed on a minimum of 50.000 events. Cells were identified based on their logarithmic side scatter dot-plot profiles. CD163 antibody was included in the kit to differentiate neutrophils from monocytes. A gate was set around the different cell populations and mean fluorescent intensity (MFI) was defined as the geometric mean of the logarithmic fluorescence intensity emitted by the respective cell lines. The calculation index of the expression CD64 value was performed by QuantiCALC software (Trillium Diagnostics, Brewer, Me, USA). CD64 indices were calculated according to the ratio of the MFI of the cell population to that of the beads. In addition, an internal negative control (lymphocyte leuko64 index < 1) and an internal positive control (monocyte leuko64 index > 3) were used to validate each sample [37]. Flow cytometry was performed within 36 h after blood sampling.

### Quantitative RT-PCR (RT-qPCR)

The total RNA from whole blood was isolated using the PAXgene blood RNA kit (Qiagen, Hilden, Germany) according to the manufacturer´s instructions. RNA of the skin lesion specimens’ was obtained using Polytron Homogenizer Model PT3100 (Kinematica AG, Switzerland) in 2 mL of TRIzol (Invitrogen, Life Technologies, USA), following the manufacturer´s instructions. After isolation, RNA was treated with DNase I (RNase-Free) (Invitrogen, Life Technologies, USA) to eliminate any genomic DNA remnants. Subsequently, RNA concentration and quality were measured via the NanoDrop 1000 spectrophotometer (Thermo Scientific, Wilmington, DE, USA). Only high-quality samples (260/280 ratios of 1.9 to 2.1) were used for the analysis. The integrity of the RNA samples was determined on a 1.2% agarose gel electrophoresis. Two-hundred nanograms and 1 μg of total RNA from blood and skin biopsies, respectively, were reverse-transcribed into complementary DNA (cDNA) using the oligo(dT) primer and SuperScriptIII (Invitrogen) following the manufacturer’s instructions. All reactions were carried out in triplicate and appropriate controls were incorporated into each run: no reverse transcriptase negative control (RT NEC) and no template negative control (RT NTC). All the reactions were incubated in StepOnePlus (Life Technologies, USA).

For blood samples, qPCR reactions were run at a final volume of 20 μL containing 250 nM of primers [human CD64 (S- GCCACAGAGGATGGAAATGT, AS- CATGAAACCAGACAGGAGTGG)], 1X Power SYBR GREEN PCR Master Mix (Thermo Scientific) and 5μL of the cDNA template. Thermal cycling conditions comprised an initial incubation at 50°C for 2 min, 95°C for 10 min, 40 cycles of denaturation at 95°C for 15 s, and annealing and extension at 60°C for 1 min. A melt curve stage was performed for each specific amplification analysis (95°C for 15 seconds, 60°C for 1 minute, and 95°C for 15 seconds). For skin biopsies, qPCR was performed using TaqMan—designed primers and probes: FCGR1A (Hs00174084_m1, Life Technologies), Glyceraldehyde-3-phosphate dehydrogenase (GAPDH; Hs99999905_m1), and TaqMan Fast Universal PCR Master Mix (2x) (Thermo Scientific) by way of the manufacturer's instructions. Real time PCR was carried out with 10 ng of cDNA and 1X of each primer in a final volume of 10 μL. Thermal cycling conditions comprised an initial incubation at 95°C for 20 s, 40 cycles of denaturation at 95°C for 1 s, and annealing and extension at 60°C for 20 s. The efficiency of each amplification reaction was calculated as the ratio between the fluorescence of the cycle of quantification and the fluorescence of the cycle immediately preceding that. The relative expression of the genes of interest was normalized by ribosomal protein L13 (RPL13; S- GACAAGAAAAAGCGGATGGT, AS- GTACTTCCAGCCAACCTCGT; Thermo Scientific) and Glyceraldehyde-3-phosphate dehydrogenase (GAPDH; Hs99999905_m1; Thermo Scientific) were used as blood and skin biopsy housekeeping genes, respectively. The expression values obtained were corrected and quantified by converting the cycle threshold (Ct) into a numerical value by using the following formula: expression value = 2-ΔCt.

### Western blot analysis

Protein extracts from frozen patient skin biopsies were obtained from archived samples following the TRIzol isolation of RNA, as previously described [38]. Twenty micrograms of protein extracts per lane were separated on 11% SDS-PAGE. After electrophoresis proteins were blotted onto nitrocellulose membranes (Bio-Rad) with a semi-dry transfer cell (Bio-Rad). Myeloperoxidase (MPO) expression was evaluated after 24 h of incubation at 4°C with a monoclonal mouse anti-human MPO heavy chain (1:100; Santa Cruz Biotechnology, catalog number SC-16128). Load control was performed using monoclonal mouse anti-human GAPDH (1:200; Santa Cruz Biotechnology, catalog number SC-47724) after 1 h of incubation at room temperature. After washing, membranes were incubated with secondary goat anti-rabbit IgG (1:2000, HRP, Dako) for 45 min at RT. After washing, bands were visualized by the chemiluminescence detection system (Western blotting Luminol Reagent; Santa Cruz Biotechnology, Inc). For densitometry analysis, the Luminol images of the developed films were performed using Adobe Photoshop CC.

### Statistical analysis

Statistical analysis was performed with GraphPad PRISM version 5 (GraphPad Software, San Diego, California, USA). Comparisons between more than 2 groups of normally distributed data were addressed via analysis of variance (ANOVA) using Bonferroni's correction for multiple testing. Differences between 2 groups were assessed by the two-tailed Student's t-test. When the data were not normally distributed, comparisons between 3 groups of variables were examined by way of the Kruskal-Wallis test with Dunn's multiple comparison post-test. To compare 2 groups of variables, the Wilcoxon was used. The adopted statistical significance level was P < 0.05. Sample size for evaluate PMN CD64 index was calculated using OpenEpi software. Significance level was established at 5% and power at 80%. The sample size required per group was 6 individuals considering the number of groups, a maximum mean difference of 2, and an expected standard deviation of 1.5. The optimal cut-off value for the PMN CD64 index was determined from receiver operating characteristic (ROC) curve analysis. The association between clinical score, number of nodules or leukogram parameters and PMN CD64 index was assessed by means of Spearman’s correlation coefficient.

## Results

### CD64 expression is modulated in ENL skin lesions subsequent to thalidomide treatment

**Fig 1:**
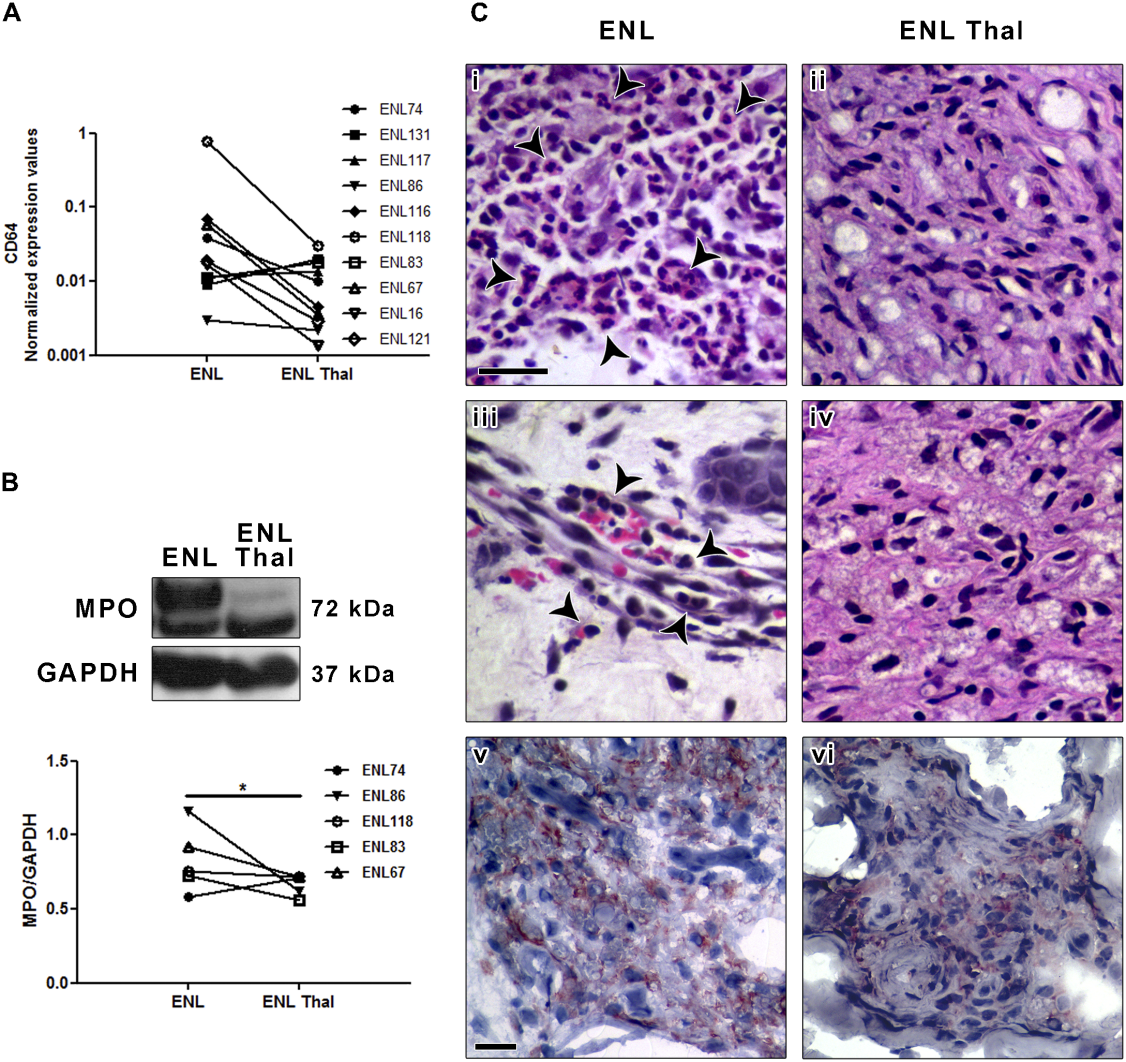
CD64 expression is modulated in ENL skin lesions after thalidomide- treatment. (A) mRNA expression of CD64 (*FCGR1A*) was assessed by qRT-PCR in ENL skin lesions at diagnosis (ENL) and 7 days after thalidomide-treatment (ENL Thal). Each line with a symbol represents a patient (n = 10). (B) Protein levels obtained from skin lesion fragments were analyzed by Western blot using antibodies against myeloperoxidase (MPO) and GAPH (loading control). The figure shows a representative Western blot analysis of 5 patients in 2 independent experiments (n = 5). The graph shows the MPO normalized values of 5 patients. Box plots show median, interquartile range, sample minimum, and maximum indications. Biopsies of ENL (Ci and Ciii) and ENL Thal (Cii and Civ) were processed for H&E. PMNs were identified with arrowheads. Scale bars = 25 μm. (Cv) ENL and ENL Thal (Cvi) skin lesions were labelled with monoclonal antibody anti-CD64. Immunoperoxidase was performed on cryosections with haematoxylin contrast. Photomicrographs are representative sections from ENL and ENL Thal (n = 6). Scale bars: 25 μm. Statistic: (A) Wilcoxon (* P < 0.05) and (B) Student's t-test (* P< 0.05).

Several studies have indicated that neutrophil CD64 expression is a highly sensitive and specific marker for systemic infection and sepsis in adults, neonates, and children [25]. In order to study whether CD64 is involved in the immunopathology of ENL, CD64 expression was analyzed via RT-qPCR in skin biopsy specimens taken at the onset of ENL episode and 7 days of thalidomide treatment (n = 10). Patient clinical data are demonstrated in S1 Table. As shown in Fig 1A, CD64 (*FCGR1a* gene) was significantly down regulated in ENL lesions after beginning thalidomide treatment. As previously reported [39], ENL patients treated with thalidomide experienced some degree of relief to total regression of their systemic symptoms with no adverse side effects. In addition, a regression of inflammatory infiltrate of ENL skin lesion was observed after starting thalidomide treatment. Although three out of ten patients had clinical and histologic improvement, but did not have the CD64 expression decreased in the lesion at the seventh day of thalidomide treatment (Fig 1A). Despite clinical and histological improvement, two of them are important to highlight. ENL121 and ENL86 patients had, respectively, maintenance and regression of ENL primary nodules regardless the emergence of new ENL inflammatory nodules.

Given that neutrophilic infiltration is a histological hallmark of ENL lesions, we evaluated whether treatment with thalidomide modulates the expression of myeloperoxidase (MPO), a major component of PMN azurophilic granules. Western blot analyses demonstrated decreased MPO levels in ENL lesions as a result of thalidomide treatment (Fig 1B). Serial sections were stained for H&E and immunohistochemical detection of CD64. ENL lesions revealed a prominent neutrophilic infiltrate distributed throughout the dermis (Fig 1Ci and 1Ciii), which, for the most part, was located in the deep dermis and around skin appendages, blood vessels, and subcutaneous adipose tissue. Intense angiogenesis, the presence of some vessels with an altered morphology, and edema in the endothelial cells and blood vessels were observed, consistent with clinical parameters of ENL. In stark contrast, the inflammatory infiltrate observed in the ENL lesions was sharply reduced after thalidomide treatment (Fig 1Cii and 1Civ). Moreover, decreased vascularization was also observed coupled with the improved clinical condition of the patients. Immunohistochemical staining for CD64 indicated the presence of CD64^+^ cells distributed within the dermis of untreated ENL patients (Fig 1Cv). On the other hand, lesions taken 7 days after thalidomide treatment (ENL Thal) were characterized by faintly-labeled cells for CD64 (Fig 1Cvi).

**Fig 2:**
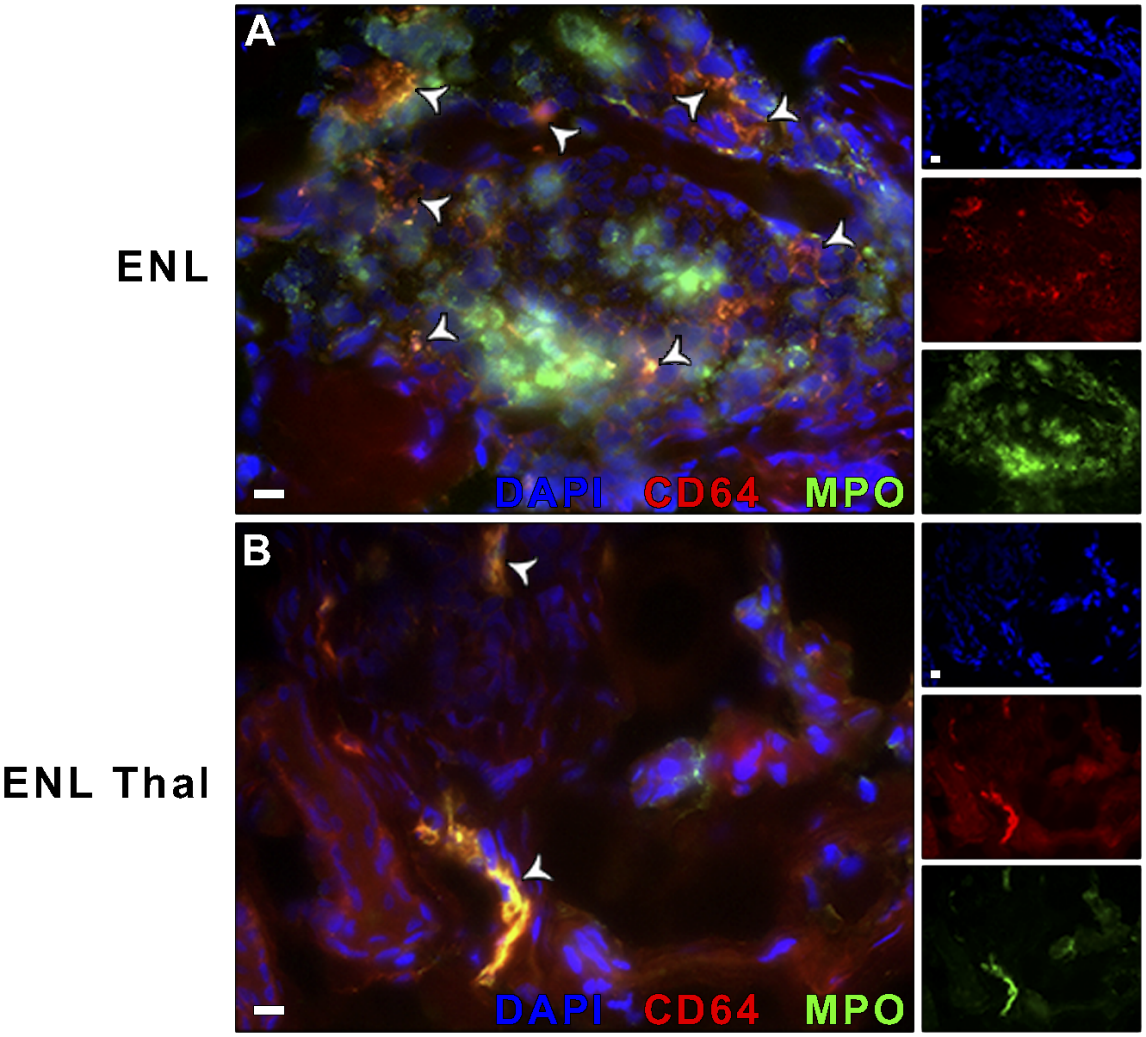
ENL patients present neutrophils expressing CD64 in skin lesions. (A) Biopsies of (A) ENL and (B) ENL thalidomide-treated patients (ENL Thal) were stained for CD64 (red), myeloperoxidase (MPO; green), and nuclei (DAPI; blue). Co-localized areas of MPO^+^CD64^+^ cells were identified with arrowheads. Right small panels represent the 3 channels. Photomicrographs are representative sections of ENL and ENL Thal (n = 3). Scale bars: 10 μm.

As a next step, we investigated if the CD64^+^ cells found in ENL lesions corresponded to MPO^+^ cells. Approximately 100% of MPO^+^ cells expressed CD64 (Fig 2A). However, the presence of other MPO^-^ cells expressing CD64, probably macrophages, could not be excluded. Lesions taken from ENL-thalidomide-treated patients revealed that MPO^+^ cells were greatly reduced. In contrast, and consistent with the presence of macrophages, CD64^+^ cells did not decrease, (Fig 2B). In summary, these data provided *in vivo* evidence that CD64^+^ neutrophils were present in the ENL lesions.

### CD64 is selectively expressed on circulating neutrophils during ENL

**Fig 3:**
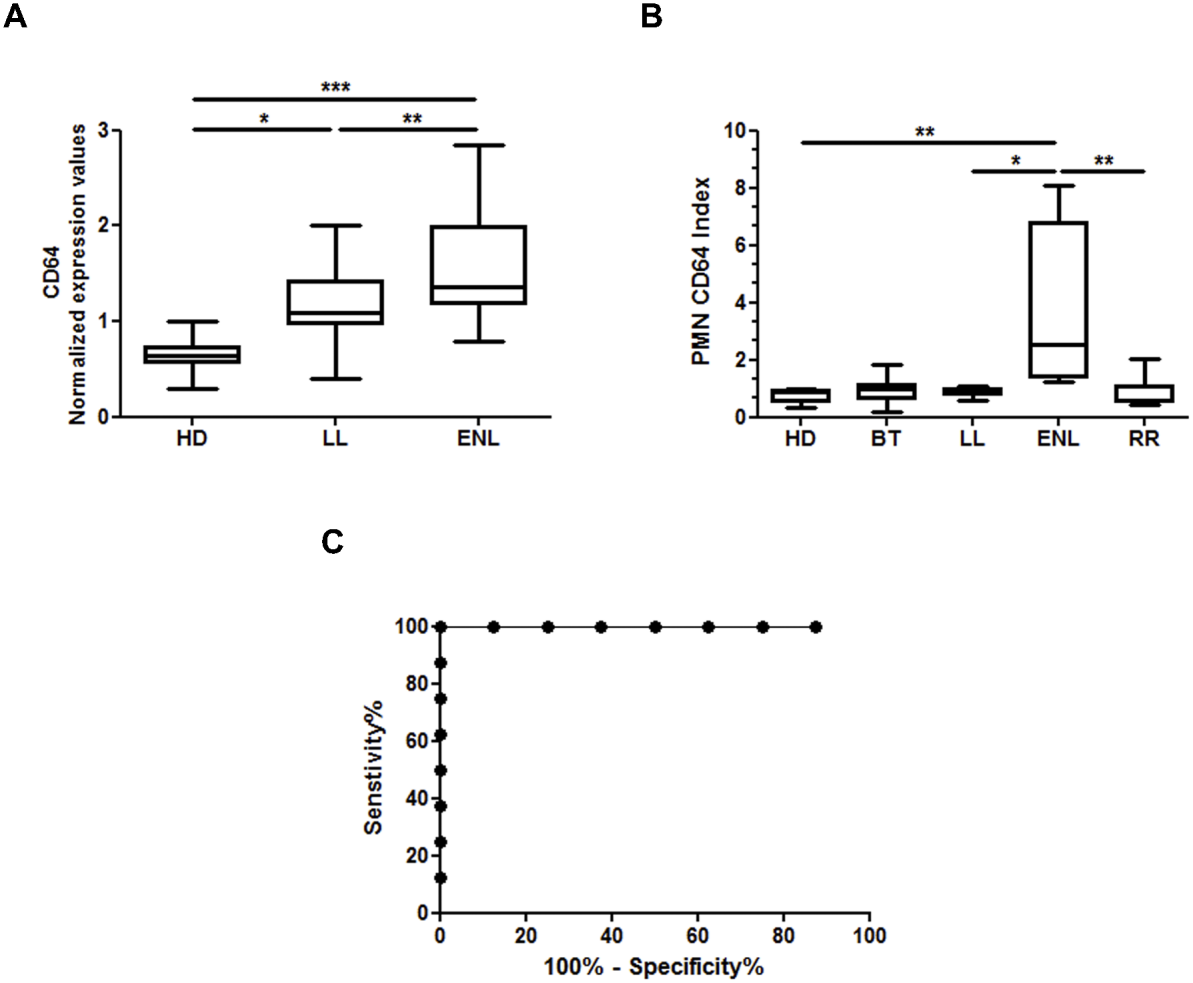
CD64 expression of CD64 on circulating neutrophils is elevated in leprosy patients with ENL. (A) mRNA expression of CD64 (*FCGR1A*) was assessed by RT-qPCR in whole blood of healthy donors (HD, n = 10), non-reactional lepromatous leprosy patients (LL, n = 8), and LL with ENL (ENL, n = 11). Box plots show median, interquartile range, sample minimum, and maximum. (B) Flow cytometry analyses of surface CD64 expression in neutrophils (PMN CD64 index) from whole blood of healthy donors (HD, n = 8), LL patients (LL, n = 8), borderline-tuberculoid leprosy (BT; n = 7), LL patients with ENL (ENL, n = 8), and reversal reaction patients (RR; n = 8). (C) Receiver operating characteristic (ROC) curves for the PMN CD64 index. Statistics: (A) ANOVA and (B) Kruskal-Wallis (* P< 0.05, ** P < 0.01 and *** P < 0.001).

Considering that up regulation of CD64 *in vivo* has been reported to be associated with the enhanced neutrophilic function [40–42], we then tested if CD64 is up regulated in ENL patients *in vivo*. The levels of CD64 gene expression in the whole blood of 8 nonreactional LL patients at diagnosis were compared before starting MDT (LL) and then compared with 11 ENL patients at diagnosis before starting thalidomide treatment (ENL). Patient clinical data are shown in S2 Table. Blood samples from 10 healthy donors (HD) were used as a control. There was a variation among the HDs, as reflected by the spread of CD64 expression (Fig 3A), likely representing a natural human variability. However, the overall expression of CD64 was significantly over‐represented in the whole blood of ENL patients as compared to that of the HDs and LL patients (Fig 3A). Moreover, CD64 expression levels were higher in LL patients when compared to the HD levels (Fig 3A). To elucidate whether the over-representation of CD64 in ENL patient blood resulted from the increased CD64 expression by a particular cell population, flow cytometric analysis of ENL blood was conducted and compared to that of HD and LL patients. To this end, CD64 expression was set using the kit Leuko64 in the whole blood of ENL, LL and HD (n = 8 / group) individuals. Patient clinical data are shown in S3 Table. An antibody, a specific monocyte marker against CD163 [43] was included to set gates for neutrophils and monocytes (S1 Fig). There were no significant differences in CD64 expression in the blood monocytes in any of the leprosy groups analysed (S2 Fig). PMN surface CD64 expression was significantly higher in ENL patients (Fig 3B). PMN CD64 index values in ENL patients were about 4.6-fold higher than among the HDs (3.564 ± 1.009 and 0.7788 ± 0.0871, respectively; P < 0.01) and about 4.1-fold higher than among LL patients (3.564 ± 1.009 and 0.8675 ± 0.05467, respectively; P < 0.01) (Fig 3B). In relation to the ENL group, circulating PMN expressed higher amounts of CD64 regardless of whether it was the first ENL reactional episode or if it occurred before, during, or after multidrug therapy (MDT) (S3 Table). In addition, there was no difference in CD64 expression between the HD and LL patients (Fig 3B). The receiver operating characteristic (ROC) curve analysis showed an area under curve of 1.0 (95% CI, 0.62–1.0; P = 0.0007834; Fig 3C). Accordingly to the ROC curve analysis, the cut-off point with the best sensitivity and specificity for PMN CD64 index was 1.115. The PMN CD64 index was above the cut-off in all ENL patients analysed (Fig 3B).

In order to analyze if CD64 is enhanced in the other clinical forms of leprosy, patients diagnosed with BT at diagnosis were recruited (n = 7). Their clinical data can be seen in S3 Table. BT neutrophils did not express surface CD64 (Fig 3B). In fact, there is no effective biomarker capable of differentiating ENL from RR, the other type of immunological leprosy reaction. Interestingly, ENL neutrophil CD64 expression was about 4.4-fold higher than among those with RR (3.563 ± 1.009 and 0.8175 ± 0.1947, respectively; P < 0.01) (Fig 3B). Thus, higher PMN CD64 index levels in leprosy patients could be predictive of an ENL outcome.

**Fig 4:**
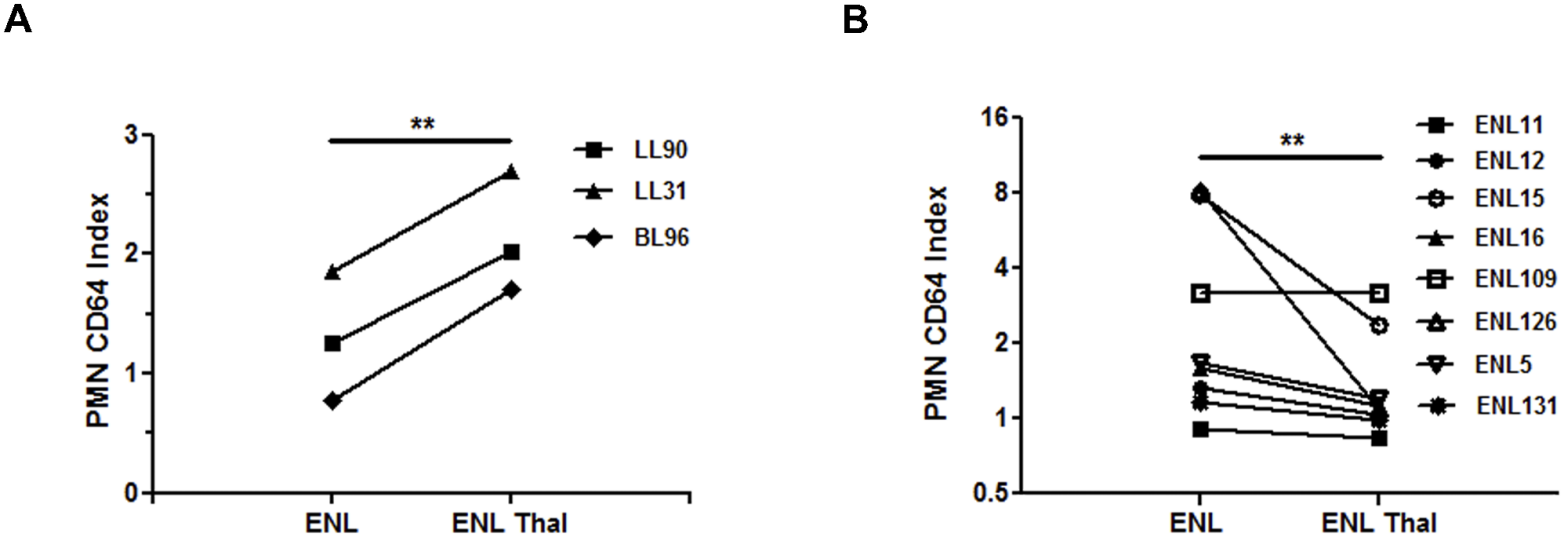
Increased expression of surface CD64 in PMN during ENL. (A) Flow cytometry analyses of surface expression of CD64 in neutrophils (PMN CD64 index) from 3 leprosy patients at the time of LL diagnosis and of the appearance of ENL (ENL). Each line with a symbol represents a patient. (B) PMN CD64 index of 8 ENL patients at diagnosis (ENL) and 7 days post-initiation of thalidomide-treatment (ENL Thal). Each line with a symbol represents a patient. Statistc: Wilcoxon (* P < 0.05 and ** P < 0.01).

It was then decided to examine whether neutrophil CD64 expression increased during ENL. Three patients were followed up at 2 sampling times: 1) At the moment of leprosy diagnosis and; 2) At the emergence of ENL. Patient clinical data are displayed in S4 Table. The neutrophil CD64 expression was enhanced in all 3 leprosy patients with the appearance of ENL (Fig 4A). It is important to note that the PMN CD64 index of the LL group was higher even before ENL (1.297 ± 0.3126), i.e., above the cut-off value of 1.115. This result requires validation by employing a much larger cohort to ascertain whether higher levels of the PMN CD64 index could effectively predict ENL. Comparative analyses of ENL patients (n = 8) before and after thalidomide treatment were performed and the resulting clinical data are demonstrated in S5 Table. PMN CD64 expression dropped after resolution (3.235 ± 1.070 and 1.474 ± 0.2940, respectively; P < 0.01) (Fig 4B). These data suggest that the PMN CD64 index declined following treatment of the reactional episode.

### Association between the high PMN CD64 index and severity of ENL

**Fig 5:**
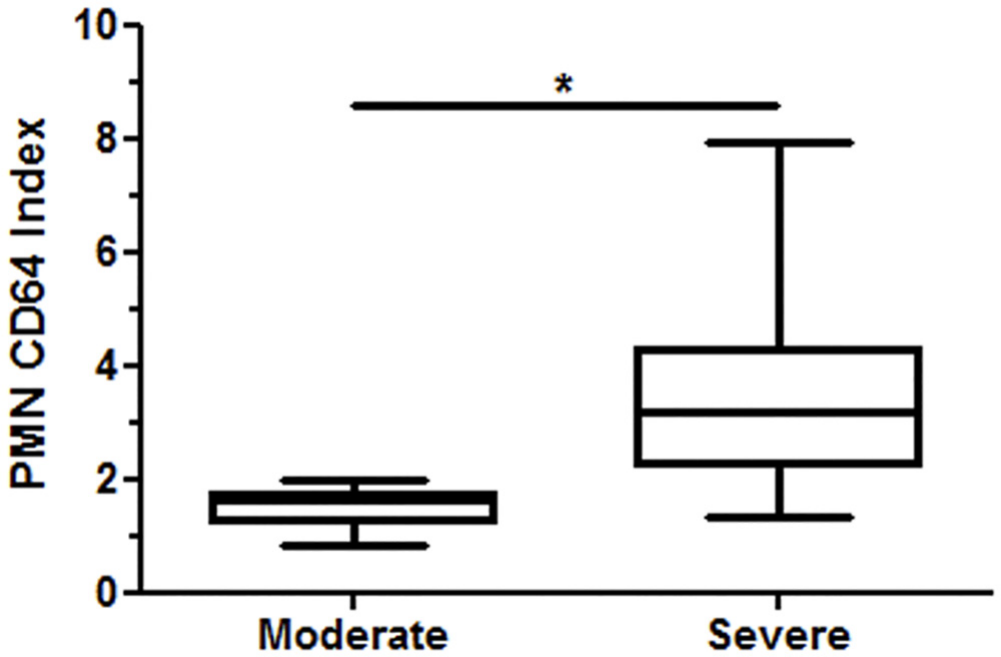
Severe ENL presents high PMN CD64 index. The PMN CD64 index was obtained by flow cytometry of whole blood. The severity scale of ENL was determined based clinical features (S7 Table). Statistic: Kruskal-Wallis (*P < 0.05).

Previous studies demonstrated that neutrophil CD64 expression is a marker of severity, and a prognosis of sepsis [28] as well as a disseminated intravascular coagulation systemic inflammatory response [29]. At this point, based on the clinical features of ENL patients, they were scored on 3 different levels: mild, moderate, and severe ENL (S6 and S7 Tables). Neutrophils from severe ENL (n = 10) presented higher levels of surface CD64 when compared to those with moderate ENL (n = 9) (Fig 5). Nine out of the 10 patients with moderate ENL had a CD64 index above the cut-off of 1.115; and the CD64 index of all 9 patients with severe ENL was actually higher than the cut-off value. The median value of the PMN CD64 index of patients with severe ENL was 2.3-fold higher than those with moderate ENL (3.542 ± 0.6451 versus 1.509 ± 0.1106, respectively; P < 0.05). The quantitative expression of CD64 showed significant correlation with the number of nodules (r = 0.4726; P = 0.0264). However, no significant correlation was observed between PMN CD64 index and temperature, localization of nodules, peripheral edema, systemic symptomatology, and leukocyte count. These results suggest that the circulating ENL CD64 neutrophil population holds the potential to induce pro-inflammatory condition.

## Discussion

In the present study, we demonstrated that CD64 is a potentially early diagnostic biomarker of ENL. CD64 gene and protein expressions were modulated via the effective treatment of ENL skin lesions with thalidomide, which was accompanied by remission of the inflammatory infiltrate. We also showed that surface CD64 expression on circulating neutrophils increased significantly during ENL and decreased after thalidomide treatment. Moreover, clinical classification of ENL patients shown higher levels of CD64 on circulating neutrophils were correlated with disease severity. These results imply that an increase in CD64 expression may be relevant to ENL.

The mechanism triggering surface expression of CD64 on circulating neutrophils needs to be further examined. We considered two possibilities concerning the increase of CD64 levels upon the appearance of ENL. Firstly, CD64 could be up regulated by inflammatory cytokines. RT-qPCR analyses performed in PBMC revealed that both IFN-γ and GM-CSF [18,19,42], formal inducers of CD64, are expressed in ENL patients but not in non-reactional patients [31]. These findings could explain the higher levels of CD64 observed exclusively in ENL patients. The second possibility is perhaps attributable to some intracellular components of *M*. *leprae*. Intact bacilli do not appear to be effective inducers of CD64. In this regard, LL and BL patients without ENL, whom presented higher amounts of bacilli, had low levels of CD64. Indeed, ENL may occur before, during or even after treatment with multidrug therapy. Rather, ENL is more prevalent during the first year of treatment in which large amounts of fragmented bacilli are found [13].

Our results strongly suggest that the PMN CD64 index is not a good parameter to detect *M*. *leprae* infection since it did not increase in patients with the non-reactional forms of the disease. Previous works have described an increased expression of CD64 in the blood PMNs in patients with bacterial infections [24,44]. However, increased CD64 expression may not occur in all bacterial infections [24]. Gram-negative bacteria also induced high surface expression of CD64 in systemic and localized infections [24]. It was previously demonstrated that the PMN of patients with active nontuberculous mycobacteria infection expressed higher levels of CD64 and this parameter was useful in monitoring active disease in rheumatoid arthritic patients [27].

Our results indicated that increased expression of CD64 is accompanied by increased ENL severity, suggesting that activation of CD64 on the surface of ENL neutrophils may contribute to the systemic inflammatory status characteristic of these patients. The data included in the present study point to a role for neutrophil CD64 in the generation of proinflammatory cytokines during ENL since ligands to CD64 [17,22,23], including immune complexes, CRP, and serum amyloid, are elevated in ENL serum [10,13,45–50]. Activation of CD64 after interaction with ligands triggers various important biological responses such as phagocytosis, endocytosis, antibody-dependent cellular cytotoxicity, release of inflammatory mediators, and enhancement of antigen presentation [20,21,51,52]. Our data point to an important role of the innate immune pathway that involves the PMN CD64 population in the inflammation that occurs during ENL. Indeed, a double-blind trial to measure the effectiveness of ENL treatment revealed that thalidomide is more effective than pentoxifylline by reducing clinical signs, systemic symptoms and laboratory parameters as such as CRP levels in the serum [39].

Analyses of circulating neutrophils demonstrated that thalidomide treatment decreased CD64 expression on neutrophils. In addition, *in situ* analysis of ENL lesions demonstrated that the neutrophils frequently found in ENL lesions and CD64 expression were likewise significantly diminished after reactional treatment. Previous studies investigating the thalidomide pathway in the context of ENL demonstrated that while monocytes stimulated *in vitro* by ligands of Toll-like receptor 2 or IgG secrete IL-8 and IL-1β the addition of thalidomide reduced the secretion of cytokines [14]. In addition, authors have demonstrated that IL-1β induces E-selectin, involved in the binding of neutrophils to endothelial cells [14].

The relationship between CD64 expression and adherence was investigated further by blocking studies using anti-CD64 antibodies. It was demonstrated that anti-CD64 antibodies prevented the binding of neutrophils of sickle cell patients in crisis to TNF-stimulated endothelium [53]. It was also shown that surface expression of CD64 enhances the interaction of neutrophils with endothelial monolayers by a factor of 7, pointing to neutrophil CD64 expression as a marker for adhesion [53]. Higher levels of CD64 in ENL PMN could be consistent with associating CD64 and the vascular damage observed in ENL lesions.

Clinically scoring ENL to define the severity of ENL and its outcome is difficult because of the multiple organ systems involved [35]. In our results, we evaluated the utility of neutrophil CD64 expression as a biomarker for assessing ENL. As a consequence, our data showed that CD64 increased in patients with severe ENL, indicating that CD64 may be an easily-accessible severity marker for ENL. We are aware that, however valuable the results, the main limitation of our study is the number of patients under consideration. It is clear that more studies need to be performed to validate the use of CD64 in clinical practice as a complementary diagnostic tool for ENL management. The prime advantage is that PMN CD64 expression could be rapidly assessed to diagnose ENL in a medical setting to ensure a more effective therapeutic decision.

## Supporting Information

S1 Fig Representative flow cytometry plots are shown for monocyte, neutrophil and lymphocyte gates. (TIF)

S2 Fig Monocyte CD64 expression in leprosy patients. (A) Flow cytometry analyses of surface CD64 expression in monocytes (MO CD64 index) from whole blood of healthy donors (HD, n = 8), non-reactional lepromatous leprosy patients (LL, n = 8), borderline-tuberculoid leprosy (BT; n = 7), LL with ENL (ENL, n = 8), and reversal reaction patients (RR; n = 8). Box plots show median, interquartile range, sample minimum, and maximum. (B) MO CD64 index from 3 leprosy patients at the time of LL diagnosis and of the appearance of ENL (ENL). Each line with a symbol represents a patient. (C) MO CD64 index of 8 ENL patients at diagnosis (ENL) and 7 days post-initiation of thalidomide-treatment (ENL Thal). Each line with a symbol represents a patient.
(TIF)

S1 Table Characteristics of patients whose skin biopsies were analyzed by RT-qPCR, western blot, and histopathology (Figs 1 and 2). C.F. = clinical form; BI = bacillary index; LL = lepromatous leprosy; MB = multibacillary leprosy; ENL = erythema nodosum leprosum; M = male; n.d = not determined; AD = at diagnosis of leprosy; AT = after treatment with multidrug therapy (MDT); DT = during treatment with MDT.
(PDF)

S2 Table Characteristics of patients whose whole blood samples were analyzed by RT-qPCR (Fig 3A). C.F. = clinical form; BI = bacillary index; LL = lepromatous leprosy; ENL = erythema nodosum leprosum; M = male; F = female; AD = at diagnosis of leprosy; AT = after treatment with MDT; DT = during treatment with multidrug therapy (MDT). *Patients whose biological samples were harvested only at diagnosis of leprosy.
(PDF)

S3 Table Characteristics of patients whose whole blood samples were analyzed by cytometry analyses (Figs 3B, 3C and S2A). C.F. = clinical form; BI = bacillary index; LL = lepromatous leprosy; BL = borderline lepromatous; BT = borderline tuberculoid; ENL = erythema nodosum leprosum; RR = reversal reaction; M = male; F = female; AD = at diagnosis of leprosy; AT = after treatment with MDT; DT = during treatment with multidrug therapy (MDT). *Patients whose biological samples were harvested at moment of leprosy diagnosis.
(PDF)

S4 Table Characteristics of patients whose whole blood samples were analyzed by cytometry analyses from LL/BL at diagnosis and at ENL diagnosis before thalidomide -treatment (Figs 4A and S2B). C.F. = clinical form; BI = bacillary index; LL = lepromatous leprosy; BL = borderline lepromatous; ENL = erythema nodosum leprosum; AD = at diagnosis of leprosy; M = male; F = female; AT = after treatment with MDT; DT = during treatment with multidrug therapy (MDT).
(PDF)

S5 Table Characteristics of patients whose whole blood samples were analyzed by cytometry analyses from the time of ENL diagnosis and 7 days post-initiation of thalidomide- treatment (ENL Thal) (Figs 4B and S2C). C.F. = clinical form; BI = bacillary index; LL = lepromatous leprosy; BL = borderline lepromatous; ENL = erythema nodosum leprosum; AD = at diagnosis of leprosy; M = male; F = female; AT = after treatment with MDT; DT = during treatment with multidrug therapy (MDT).
(PDF)

S6 Table Characteristics of patients whose clinical data were used to create the ENL severity scale (Fig 5). C.F. = clinical form; BI = bacillary index; LL = lepromatous leprosy; BL = borderline lepromatous; ENL = erythema nodosum leprosum; AD = at diagnosis of leprosy; M = male; F = female; AT = after treatment with multidrug therapy (MDT); DT = during treatment with MDT.
(PDF)

S7 Table Clinical features of ENL. SS = Systemic symptomatology. ^1^Score: 0 = 37.5°C or less; 1 = No fever at the moment but reported fever in the last 7 days; 2 = 37.6–38.5°C; 3 = 38.6 or more. ^2^Score: 0 = 0; 1 = 1–10; 2 = 11–20; 3 = 21 or more. ^3^Score: 0 = absent; 1 = 1–2 regions; 2 = 3–4 regions; 3 = 5–7 regions. ^4^Score: 0 = absent; 1 = 1 site of hands or feet or face; 2 = 2 sites; 3 = All 3 sites (hands, feet and face). ^5^Score: 0 = absent, 1 = 1 systemic symptom; 2 = 2 systemic symptons; 3 = 3 or more systemic symptons.
(PDF)

S1 Checklist STROBE checklist. (DOC)
