# Supplementary figures and images for: Expression of CD64 on Circulating Neutrophils Favoring Systemic Inflammatory Status in Erythema Nodosum Leprosum
Source: PLoS Negl Trop Dis. 2016 Aug 24;10(8):e0004955. doi: 10.1371/journal.pntd.0004955 (PMC4996526; doi:10.1371/journal.pntd.0004955)

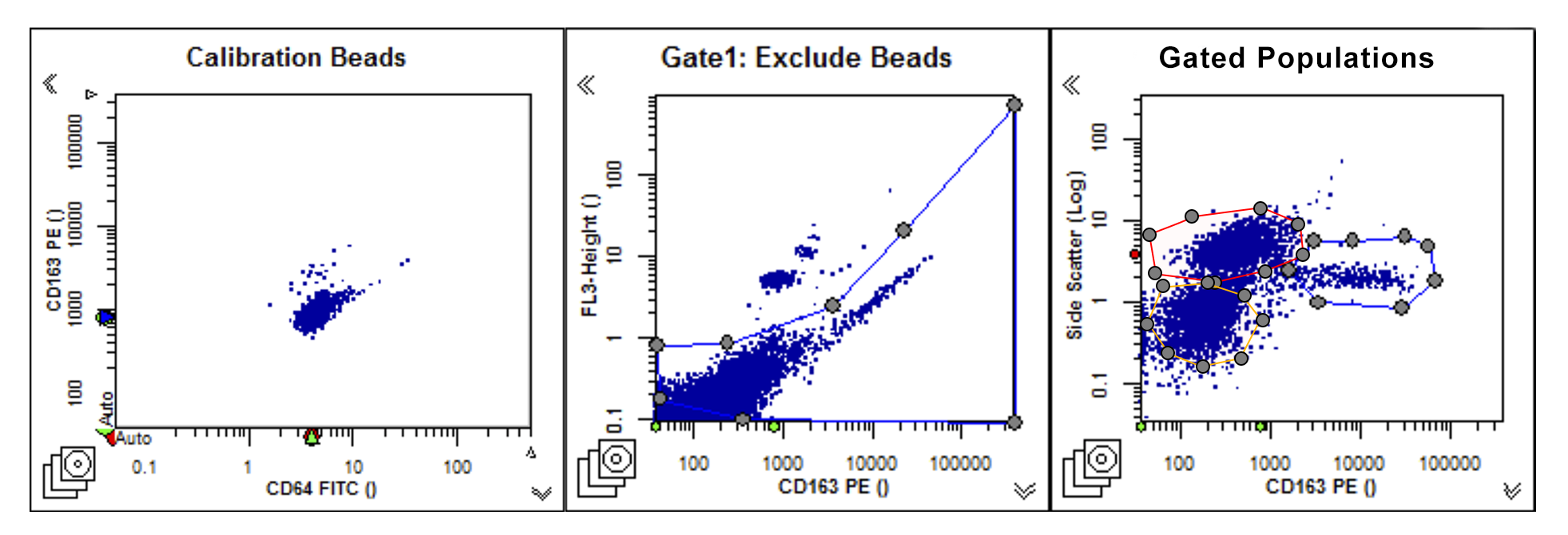

Supplement: S1 Fig — (TIF) [file pntd.0004955.s001.tif]

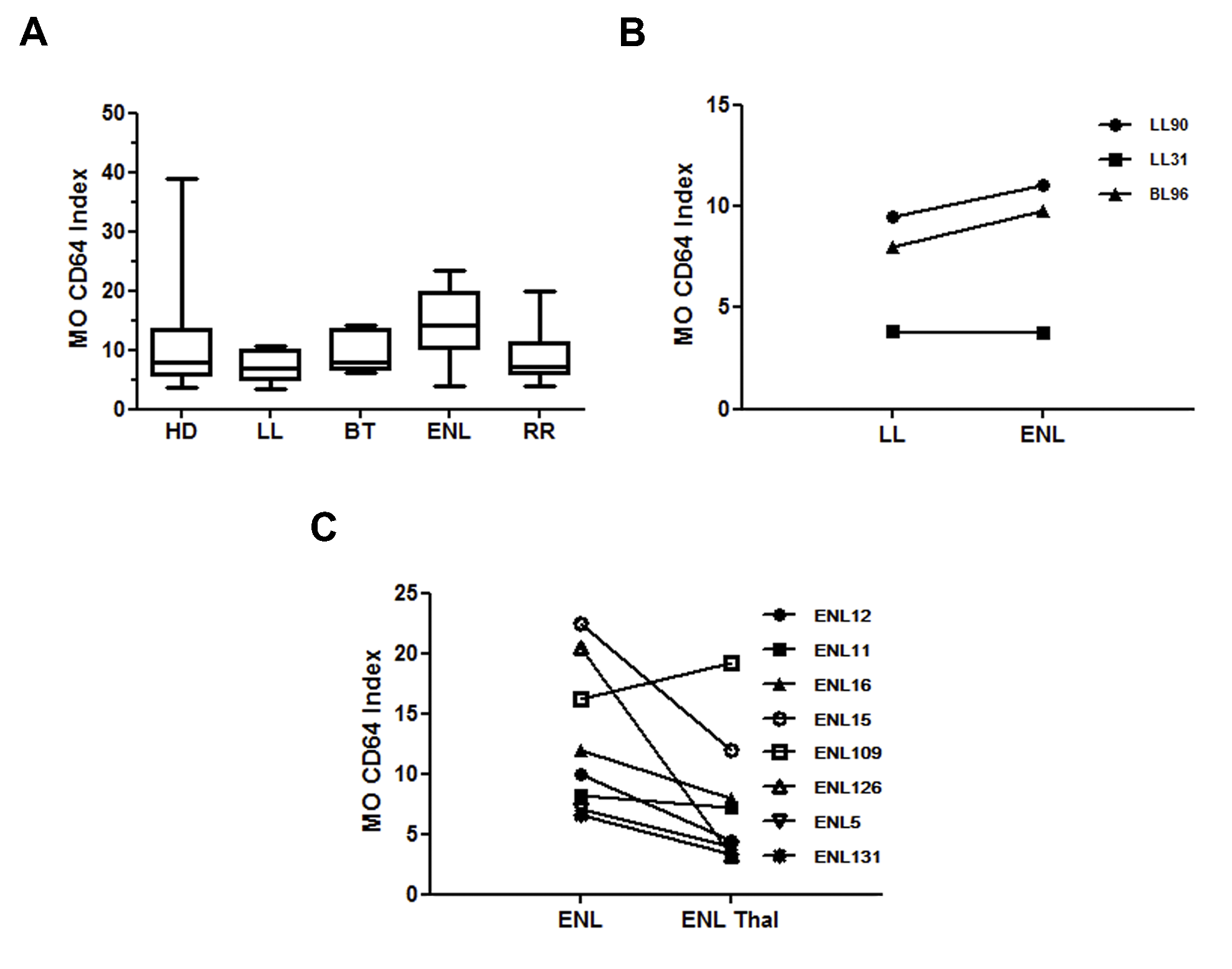

Supplement: S2 Fig — (A) Flow cytometry analyses of surface CD64 expression in monocytes (MO CD64 index) from whole blood of healthy donors (HD, n = 8), non-reactional lepromatous leprosy patients (LL, n = 8), borderline-tuberculoid leprosy (BT; n = 7), LL with ENL (ENL, n = 8), and reversal reaction patients (RR; n = 8). Box plots show median, interquartile range, sample minimum, and maximum. (B) MO CD64 index from 3 leprosy patients at the time of LL diagnosis and of the appearance of ENL (ENL). Each line with a symbol represents a patient. (C) MO CD64 index of 8 ENL patients at diagnosis (ENL) and 7 days post-initiation of thalidomide-treatment (ENL Thal). Each line with a symbol represents a patient. (TIF) [file pntd.0004955.s002.tif]
