# Supplementary material for: Expression of CD64 on Circulating Neutrophils Favoring Systemic Inflammatory Status in Erythema Nodosum Leprosum
Source: PLoS Negl Trop Dis. 2016 Aug 24;10(8):e0004955. doi: 10.1371/journal.pntd.0004955 (PMC4996526; doi:10.1371/journal.pntd.0004955)
Supplement: S1 Table — C.F. = clinical form; BI = bacillary index; LL = lepromatous leprosy; MB = multibacillary leprosy; ENL = erythema nodosum leprosum; M = male; n.d = not determined; AD = at diagnosis of leprosy; AT = after treatment with multidrug therapy (MDT); DT = during treatment with MDT. (PDF) [file pntd.0004955.s003.pdf]

S1 Table

Patient code	Sex	Age	C.F.	BI	Reaction type	Reaction diagnosis	First episode
ENL74	M	46	LL	5.9	ENL	AT	Yes
ENL131	M	62	BL	3.5	ENL	AT	No
ENL117	M	41	LL	5.3	ENL	AD	Yes
ENL86	M	30	LL	5.8	ENL	DT	Yes
ENL116	M	19	LL	4.85	ENL	AT	Yes
ENL118	M	51	LL	4.6	ENL	AT	No
ENL83	M	20	LL	5.85	ENL	DT	Yes
LL67	M	17	LL	4.85	ENL	AT	No
ENL16	M	23	LL	5	ENL	DT	Yes
ENL121	M	27	MB	n.d	ENL	AT	n.d.

Characteristics of patients whose skin biopsies were analyzed by RT-qPCR, Western blot, and histopathology (Fig. 1 and 2). C.F. = clinical form; BI = bacillary index; LL = lepromatous leprosy; MB = multibacillary leprosy; ENL = erythema nodosum leprosum; M = male; n.d = not determined; AD = at diagnosis of leprosy; AT = after treatment with multidrug therapy (MDT); DT = during treatment with MDT.
