# Supplementary material for: Expression of CD64 on Circulating Neutrophils Favoring Systemic Inflammatory Status in Erythema Nodosum Leprosum
Source: PLoS Negl Trop Dis. 2016 Aug 24;10(8):e0004955. doi: 10.1371/journal.pntd.0004955 (PMC4996526; doi:10.1371/journal.pntd.0004955)
Supplement: S2 Table — C.F. = clinical form; BI = bacillary index; LL = lepromatous leprosy; ENL = erythema nodosum leprosum; M = male; F = female; AD = at diagnosis of leprosy; AT = after treatment with MDT; DT = during treatment with multidrug therapy (MDT). *Patients whose biological samples were harvested only at diagnosis of leprosy. (PDF) [file pntd.0004955.s004.pdf]

S2 Table

Patient code	Sex	Age	C.F.	BI	Reaction type	Reaction diagnosis	First episode
LL77	F	60	LL	5.95	*	-	-
LL71	M	32	LL	5.85	*	-	-
LL81	F	53	LL	5.9	*	-	-
LL82	M	46	LL	4.85	*	-	-
LL74	M	46	LL	5.9	*	-	-
LL105	F	64	LL	4.85	*	-	-
LL83	M	20	LL	5.85	*	-	-
LL107	F	31	LL	4.6	*	-	-
ENL60	F	47	LL	3.7	ENL	AT	No
ENL62	M	36	LL	3.6	ENL	AD	Yes
ENL75	M	43	LL	5.8	ENL	AT	No
ENL2	M	50	LL	5.85	ENL	AT	No
ENL63	M	30	LL	3.7	ENL	AD	Yes
ENL50	M	57	LL	5.8	ENL	AT	No
ENL46	M	55	LL	5.85	ENL	DT	Yes
ENL69	M	39	LL	4.85	ENL	DT	Yes
ENL67	M	17	LL	4.85	ENL	AT	No
ENL36	M	57	LL	3.7	ENL	AD	Yes

Characteristics of patients whose whole blood samples were analyzed by RT-qPCR (Fig. 3A). C.F. = clinical form; BI = bacillary index; LL = lepromatous leprosy; ENL = erythema nodosum leprosum; M = male; F = female; AD = at diagnosis of leprosy; AT

= after treatment with MDT; DT = during treatment with multidrug therapy (MDT).

*Patients whose biological samples were harvested only at diagnosis of leprosy.
