# Supplementary material for: Expression of CD64 on Circulating Neutrophils Favoring Systemic Inflammatory Status in Erythema Nodosum Leprosum
Source: PLoS Negl Trop Dis. 2016 Aug 24;10(8):e0004955. doi: 10.1371/journal.pntd.0004955 (PMC4996526; doi:10.1371/journal.pntd.0004955)
Supplement: S3 Table — C.F. = clinical form; BI = bacillary index; LL = lepromatous leprosy; BL = borderline lepromatous; BT = borderline tuberculoid; ENL = erythema nodosum leprosum; RR = reversal reaction; M = male; F = female; AD = at diagnosis of leprosy; AT = after treatment with MDT; DT = during treatment with multidrug therapy (MDT). *Patients whose biological samples were harvested at moment of leprosy diagnosis. (PDF) [file pntd.0004955.s005.pdf]

S3 Table

Patient code	Sex	Age	C.F.	BI in	Reaction type	Reaction diagnosis	First episode
LL77	F	60	LL	5.95	*	-	-
LL71	M	32	LL	5.85	*	-	-
LL74	M	46	LL	5.9	*	-	-
LL32	F	21	LL	5.95	*	-	-
LL87	M	38	LL	5.7	*	-	-
LL143	M	45	LL	5.95	*	-	-
LL138	M	61	LL	4.85	*	-	-
LL129	M	44	LL	5.85	*	-	-
ENL2	M	50	LL	5.85	ENL	AT	No
ENL12	M	35	LL	4.8	ENL	DT	Yes
ENL15	F	68	LL	5.85	ENL	AT	No
ENL16	M	23	LL	5	ENL	DT	Yes
ENL109	M	31	LL	4.5	ENL	AD	Yes
ENL126	M	37	LL	3.5	ENL	AT	Yes
ENL31	M	35	LL	4.85	ENL	DT	Yes
ENL111	F	38	LL	4.5	ENL	DT	Yes
RR113	M	60	BL	4.7	RR	DT	Yes
RR17	M	35	BL	3.5	RR	AD	Yes
RR125	M	35	BL	3.5	RR	AT	Yes
RR13	F	58	BT	0	RR	AD	Yes
RR30	F	45	BT	0	RR	DT	Yes
RR52	F	56	BT	0	RR	AD	Yes

RR64	M	28	BT	0	RR	AD	Yes
RR49	M	72	BL	4.5	RR	AT	Yes
BT135	F	35	BT	0	*	-	-
BT53	F	50	BT	0	*	-	-
BT91	F	18	BT	0	*	-	-
BT92	M	57	BT	0	*	-	-
BT93	F	35	BT	0	*	-	-
BT94	F	35	BT	0	*	-	-
BT97	M	60	BT	0	*	-	-

Characteristics of patients whose whole blood samples were analyzed by cytometry analyses (Fig. 3B,C and S2 Fig. A). C.F. = clinical form; BI = bacillary index; LL= lepromatous leprosy; BL = borderline lepromatous; BT = borderline tuberculoid; ENL = erythema nodosum leprosum; RR = reversal reaction; M = male; F = female; AD = at diagnosis of leprosy; AT = after treatment with MDT; DT = during treatment with multidrug therapy (MDT). *Patients whose biological samples were harvested at moment of leprosy diagnosis.
