# Supplementary material for: Expression of CD64 on Circulating Neutrophils Favoring Systemic Inflammatory Status in Erythema Nodosum Leprosum
Source: PLoS Negl Trop Dis. 2016 Aug 24;10(8):e0004955. doi: 10.1371/journal.pntd.0004955 (PMC4996526; doi:10.1371/journal.pntd.0004955)
Supplement: S4 Table — C.F. = clinical form; BI = bacillary index; LL = lepromatous leprosy; BL = borderline lepromatous; ENL = erythema nodosum leprosum; AD = at diagnosis of leprosy; M = male; F = female; AT = after treatment with MDT; DT = during treatment with multidrug therapy (MDT). (PDF) [file pntd.0004955.s006.pdf]

S4 Table

Patient code	Sex	Age	C.F.	BI	Reaction type	Reaction diagnosis	First episode
LL90	M	45	LL	5.75	ENL	AT	Yes
LL31	M	35	LL	4.85	ENL	DT	Yes
BL96	M	31	BL	3.3	ENL	DT	Yes

Characteristics of patients whose whole blood samples were analyzed by cytometry analyses from LL/BL at diagnosis and at ENL diagnosis before thalidomide - treatment (Fig. 4A and S2 Fig. B). C.F. = clinical form; BI = bacillary index; LL= lepromatous leprosy; BL = borderline lepromatous; ENL = erythema nodosum leprosum; AD = at diagnosis of leprosy; M = male; F = female; AT = after treatment with MDT; DT = during treatment with multidrug therapy (MDT).
