# Supplementary material for: Expression of CD64 on Circulating Neutrophils Favoring Systemic Inflammatory Status in Erythema Nodosum Leprosum
Source: PLoS Negl Trop Dis. 2016 Aug 24;10(8):e0004955. doi: 10.1371/journal.pntd.0004955 (PMC4996526; doi:10.1371/journal.pntd.0004955)
Supplement: S5 Table — C.F. = clinical form; BI = bacillary index; LL = lepromatous leprosy; BL = borderline lepromatous; ENL = erythema nodosum leprosum; AD = at diagnosis of leprosy; M = male; F = female; AT = after treatment with MDT; DT = during treatment with multidrug therapy (MDT). (PDF) [file pntd.0004955.s007.pdf]

S5 Table

Patient code	Sex	Age	C.F.	BI	Reaction type	Reaction diagnosis	First episode
ENL11	M	62	LL	5	ENL	AT	Yes
ENL12	M	35	LL	4.8	ENL	DT	Yes
ENL15	F	68	LL	5.85	ENL	AT	No
ENL16	M	23	LL	5	ENL	DT	Yes
ENL109	M	31	LL	4.5	ENL	AD	Yes
ENL126	M	37	LL	3.5	ENL	AT	Yes
ENL5	M	57	LL	5.25	ENL	AT	No
ENL131	M	63	BL	3.5	ENL	AT	No

Characteristics of patients whose whole blood samples were analyzed by cytometry analyses from the time of ENL diagnosis and 7 days post-initiation of thalidomide-treatment (ENL Thal) (Fig. 4B and and S2 Fig. C). C.F. = clinical form; BI = bacillary index; LL= lepromatous leprosy; BL = borderline lepromatous; ENL = erythema nodosum leprosum; AD = at diagnosis of leprosy; M= male; F = female; AT = after treatment with MDT; DT = during treatment with multidrug therapy (MDT).
