# Supplementary material for: Expression of CD64 on Circulating Neutrophils Favoring Systemic Inflammatory Status in Erythema Nodosum Leprosum
Source: PLoS Negl Trop Dis. 2016 Aug 24;10(8):e0004955. doi: 10.1371/journal.pntd.0004955 (PMC4996526; doi:10.1371/journal.pntd.0004955)
Supplement: S6 Table — C.F. = clinical form; BI = bacillary index; LL = lepromatous leprosy; BL = borderline lepromatous; ENL = erythema nodosum leprosum; AD = at diagnosis of leprosy; M = male; F = female; AT = after treatment with multidrug therapy (MDT); DT = during treatment with MDT. (PDF) [file pntd.0004955.s008.pdf]

S6 Table

Patient code	Sex	Age	C.F.	BI	Reaction type	Reaction diagnosis	First episode
ENL67	M	17	LL	4.85	ENL	AT	No
ENL90	M	45	LL	5.5	ENL	AT	Yes
ENL2	M	50	LL	5.85	ENL	AT	No
ENL12	M	35	LL	4.8	ENL	DT	Yes
ENL15	F	68	LL	5.85	ENL	AT	No
ENL16	M	23	LL	5	ENL	DT	Yes
ENL37	M	28	LL	5.5	ENL	AT	Yes
ENL46	M	55	LL	5.85	ENL	DT	Yes
ENL50	M	57	LL	5.8	ENL	AT	No
ENL69	M	39	LL	4.85	ENL	DT	Yes
ENL63	M	30	LL	3.7	ENL	AD	Yes
ENL75	M	43	LL	5.8	ENL	AT	No
ENL76	M	58	LL	4.85	ENL	AT	No
ENL109	M	31	LL	4.5	ENL	AD	Yes
ENL108	M	61	LL	5.95	ENL	DT	Yes
ENL110	M	22	LL	4.8	ENL	DT	Yes
ENL111	F	38	LL	4.5	ENL	DT	Yes
ENL112	M	45	LL	4.9	ENL	DT	Yes
ENL114	M	29	LL	4.6	ENL	AT	Yes
ENL126	M	37	LL	3.5	ENL	AT	No
ENL7	M	20	BL	2.75	ENL	AT	No
ENL96	M	32	BL	3.3	ENL	DT	Yes

Characteristics of patients whose clinical data were used to create the ENL severity scale (Fig. 5). C.F. = clinical form; BI = bacillary index; LL= lepromatous leprosy; BL = borderline lepromatous; ENL = erythema nodosum leprosum; AD = at diagnosis of leprosy; M= male; F = female; AT = after treatment with multidrug therapy (MDT); DT = during treatment with MDT.
