# Supplementary material for: Expression of CD64 on Circulating Neutrophils Favoring Systemic Inflammatory Status in Erythema Nodosum Leprosum
Source: PLoS Negl Trop Dis. 2016 Aug 24;10(8):e0004955. doi: 10.1371/journal.pntd.0004955 (PMC4996526; doi:10.1371/journal.pntd.0004955)
Supplement: S7 Table — SS = Systemic symptomatology. 1Score: 0 = 37.5°C or less; 1 = No fever at the moment but reported fever in the last 7 days; 2 = 37.6–38.5°C; 3 = 38.6 or more. 2Score: 0 = 0; 1 = 1–10; 2 = 11–20; 3 = 21 or more. 3Score: 0 = absent; 1 = 1–2 regions; 2 = 3–4 regions; 3 = 5–7 regions. 4Score: 0 = absent; 1 = 1 site of hands or feet or face; 2 = 2 sites; 3 = All 3 sites (hands, feet and face). 5Score: 0 = absent, 1 = 1 systemic symptom; 2 = 2 systemic symptons; 3 = 3 or more systemic symptons. (PDF) [file pntd.0004955.s009.pdf]

S7 Table

Patient code	Temperature[1]	Number of nodules[2]	Localization of nodules[3]	Peripheral edema[4]	SS[5]	Leukogram (Total cells/mm^3)	ENL classification	PMN CD64 index
ENL67	3	2	1	0	3	20250	Moderate	1.81
ENL90	0	3	1	2	3	13770	Severe	2.02
ENL2	0	3	3	1	3	16040	Severe	3.28
ENL12	0	3	1	0	3	11530	Severe	1.32
ENL15	3	3	1	1	3	13350	Severe	7.92
ENL16	0	1	1	0	3	7680	Moderate	1.60
ENL37	0	3	3	2	3	n.d.	Severe	4.93
ENL46	2	3	3	2	2	11570	Severe	2.40

ENL50	0	3	1	0	0	9040	Mild	1.38
ENL69	0	1	1	1	2	6330	Moderate	1.72
ENL63	0	1	2	3	3	12440	Moderate	1.39
ENL75	0	3	2	0	3	8370	Moderate	1.64
ENL76	0	3	1	2	3	18490	Severe	3.62
ENL109	2	3	1	0	3	13780	Severe	3.20
ENL108	3	3	3	2	3	4730	Moderate	1.17
ENL110	0	3	1	1	3	n.d.	Moderate	1.99
ENL111	2	3	1	3	3	7040	Moderate	1.23
ENL112	0	1	1	0	1	n.d.	Mild	0.91
ENL114	0	3	3	0	3	n.d.	Severe	3.19

ENL126	1	3	3	0	3	n.d.	Severe	8.10
ENL7	0	1	1	2	3	n.d.	Moderate	0.83
ENL96	1	1	2	0	3	12070	Moderate	1.71

Clinical features of ENL. Legend: SS= Systemic symptomatology. [1]Score: 0 = 37.5^{o} C or less; 1 = No fever at the moment but reported fever in the last 7 days; 2 = 37.6-38.5^{o} C; 3 = 38.6 or more. [2]Score: 0 = 0; 1= 1-10; 2= 11-20; 3 = 21 or more. [3]Score: 0 = absent; 1 = 1-2 regions; 2 = 3-4 regions; 3 = 5-7 regions. [4]Score: 0 = absent; 1 = 1 site of hands or feet or face; 2 = 2 sites; 3 = All 3 sites (hands, feet and face). [5]Score: 0 = absent, 1 = 1 systemic symptom; 2 = 2 systemic symptons; 3 = 3 or more systemic symptons.
